# From crisis to crisis: impacts of the COVID-19 pandemic on people living with HIV and HIV/AIDS service organizations in Indiana

**DOI:** 10.1186/s12913-022-07998-0

**Published:** 2022-05-09

**Authors:** Justin J. MacNeill, Jacqueline C. Linnes, Randolph D. Hubach, Natalia M. Rodriguez

**Affiliations:** 1 Weldon School of Biomedical Engineering, College of Engineering, Purdue University, IN West Lafayette, USA; 2 Department of Public Health, College of Health and Human Sciences, Purdue University, IN West Lafayette, USA

**Keywords:** HIV, COVID-19, Socio-ecological model, AIDS service organizations

## Abstract

**Background:**

The COVID-19 pandemic thrust people living with HIV (PLWH) and HIV/AIDS service organizations into an environment ripe with uncertainty. This study examined Indiana HIV/AIDS service provider perceptions of how COVID-19 affected the overall health and access to care of their clients, and how the organizations prepared for, adapted, and responded to the needs of PLWH during the pandemic.

**Methods:**

Guided by the socioecological model, fifteen semi-structured interviews were conducted with ten different HIV/AIDS service organizations across the state of Indiana.

**Results:**

Despite the profound disruptions experienced by HIV programs, HIV/AIDS service organizations responded quickly to the challenges posed by the COVID-19 pandemic through myriad innovative strategies, largely informed by prior experiences with the HIV epidemic.

**Conclusions:**

The lessons provided by HIV/AIDS service organizations are invaluable to informing future pandemic response for PLWH. Service delivery innovations in response to the COVID-19 crisis may provide insights to improve HIV care continuity strategies for vulnerable populations far beyond the pandemic.

**Supplementary Information:**

The online version contains supplementary material available at 10.1186/s12913-022-07998-0.

## Background

The Human immunodeficiency virus (HIV) is a persistent public health crisis in the United States, especially among medically-underserved populations including racial and ethnic minorities [1], as well as people who use drugs (PWUD) who are 22 times more likely to acquire HIV than the average population [2]. As of 2018, 1.2 million existing cases of HIV, along with 38,000 new annual diagnoses were documented in the United States [1].

The onset of COVID-19 precipitated an especially precarious environment for people living with HIV (PLWH). Not only has COVID-19 increased the difficulty of managing HIV care, but existing comorbidities prevalent within the HIV community have meant high susceptibility to COVID-19 and adverse outcomes [3]. PLWH have also been found to be at a potentially higher risk of SARS-CoV-2 infection, in part due to higher rates of substance use, violence, stigma, discrimination, poverty and homelessness within the population [4–8]. The interactions between COVID-19, HIV, and other risk factors has been evaluated through a syndemic perspective in which COVID-19 is assumed to function in tandem with a myriad of health challenges faced by PLWH and is influenced by proximal and distal factors.

Social and economic inequality drive syndemics [8–10]. Homelessness and congregate living among PLWH has been further exacerbated by employment and income loss due to residual economic effects of COVID-19 and, at times, has been precipitated by a COVID-19 infection [11]. Income loss also contributed to increased food insecurity among PLWH, particularly among younger individuals; US studies showed around 40% of PLWH respondents reported food insecurity, with that rate increasing to 84% among people of color [12, 13]. Food insecurity is especially dangerous for PLWH because it has the potential to induce deleterious downstream effects within HIV management, including antiretroviral therapy (ART) non-adherence and decreased clinic visits [12]. In addition, both fear of and active COVID-19 coinfection were indicated to aggravate existing mental health struggles due to additional stress and isolation [14, 15].

By June 2020, 13% of Americans reported that they had started using, or had increased substance use, in order to cope with stress and anxiety related to COVID-19 and many communities are seeing the overlapping effects of the COVID-19 crisis with the ongoing opioid crisis in the U.S. [16]. Concurrently, social and environmental factors associated with drug use may increase the risk of COVID-19 infection, especially among individuals who experience homelessness, which is common among those with a substance use disorder [17]. PWUD also face increased risk for adverse health outcomes overall, due to shortages and other barriers to obtaining medications, drugs, syringe exchange services, and overall health care. This is particularly worrisome due to the ongoing high risk of HIV among PWUD, as COVID-related disruptions in access to healthcare meant less access to HIV testing and less linkages to care for those who test positive for HIV.

HIV and AIDS service organizations around the nation were tasked with addressing multiple barriers to care and social determinants (e.g., housing, travel to medical visits, social support) exacerbated by COVID-19 with the additional service disruptions and limitations due to pandemic and associated public health protocols. However, prior experiences with the HIV epidemic were instrumental in responding and ensuring communities continue to access HIV prevention services [18, 19]. Numerous studies reported substantially reduced access and utilization of pre-exposure prophylaxis (PrEP), initial PrEP intake visits, and increases in refill lapses during the COVID-19 pandemic [20, 21]. Many US cities also reported significant decreases in HIV testing rates – San Francisco, for example, reporting a 40% decrease citywide and a 90% decrease in community-based testing [20]. PLWH also personally voiced concerns about limited on-site and at-home testing, and over a fifth of respondents to a global survey stated they lost access to their HIV provider, with the uninsured more likely to lose access [22]. Moreover, the introduction of shelter-in-place policies necessitated the onset of virtual outpatient medical management, and although virtual attendance rates generally mirrored pre-pandemic in-person attendance rates, the transition to telehealth coincided with decreased viral suppression rates, especially among homeless PLWH [23, 24]. A South Carolina study outlined services provided through telehealth which included client intake, non-medical case management, support groups, medication adherence assessments, and medication refills, among others. Barriers to telehealth adoption and implementation were present on both the provider and client ends. Low-resource organizations struggled to afford the technological infrastructure needed to establish efficiency and security. Clients reported a lack of smartphone access and digital literacy needed for video calls or app-based visits as well as some discomfort due to a lack of patient-provider intimacy [25].

In Indiana, over 11,500 individuals living with HIV were documented in 2018 at a rate of 206 cases per 100,000 people, approximately 55% of the national rate and in-line with the regional rate [26] Cases are primarily among White individuals, but HIV rates among Black and Hispanic individuals remain disproportionate compared to their population size in the state [26]. Indiana is reflective of other states in the US in which the HIV epidemic has changed with a shift towards rural areas where populations are dispersed, and health care resources are limited [27, 28]. Concurrent socioeconomic factors, geography, and cultural context are coalescing for populations living in rural communities in a way that places individuals at risk for HIV infection [29, 30]. In rural Indiana, the abuse of and dependence on opiates led to the use of injectable opioids or heroin. In the absence of needle-exchange programs, many had little choice but to share and an HIV outbreak ensued [31]. Unlike other outbreaks among people who inject drugs, most people diagnosed in the Indiana outbreak live in rural communities and are young (median age, 32 years; range, 18 to 57) and White, and almost half are women [32].

Prior to COVID-19, of those in Indiana living with HIV, 77% reported receiving medical care, and 62% were documented as virally suppressed [26]. Twelve primary HIV/AIDS service organizations office, 23 total sites including satellite locations, around the state of Indiana provide PLWH with non-medical case management services. HIV/AIDS service organizations are the primary implementers of HIV care services in Indiana. Previous work, conducted just prior to COVID-19, with HIV social service providers in Indiana has documented the experiences of this cadre of health professionals, including a lack of perceived control over systems to support PLWH clients and constant occupational stressors [19]. The impact of COVID-19 on an already strained HIV workforce and agencies has not been explored in relatively rural states, like Indiana.

Guided by the socioecological model (SEM) [33], which recognizes the interrelatedness of person-environment, this study examined Indiana-based HIV/AIDS service provider perceptions of how COVID-19 affected the overall health and access to care of their PLWH clients, and how the organizations prepared for, adapted, and responded to the needs of PLWH during the pandemic. Understanding these individual- and organizational-level challenges and responses can be instrumental to informing HIV service delivery innovations moving forward, as well as future pandemic response for vulnerable populations.

## Methods

Guided by the SEM, with a focus on the interrelatedness of factors at the individual, interpersonal, organizational, community, and public policy levels that influence health behaviors and outcomes, an initial interview guide was developed to understand the multilevel challenges and responses to supporting PLWH during the COVID-19 pandemic, from the perspective of Indiana HIV/AIDS service organizations. The final interview guide included questions such as: “Under normal circumstances (pre-COVID), what specific services or resources does your organization provide for individuals with HIV?”, “Did COVID change service provision in any way for your organization?”, “In your view, how has the COVID-19 pandemic impacted your clients?”, and “Did COVID-related policy changes affect your organization or operations?”. The interview guide developed for this study is provided as Additional file 1.

To obtain the most comprehensive accounting of individual and organizational HIV-related activity during the COVID-19 pandemic, staff members of the 12 major HIV/AIDS service organizations serving PLWH from around Indiana were recruited for interviews. Recruitment occurred via snowball sampling as follows. First, initial contact was made with a representative of an HIV/AIDS service organization who provided a comprehensive list of all HIV/AIDS service organizations and testing centers in the state. Then, from September 2020 through November 2020, individuals with publicly-available email addresses from each of the organizations were sent via email, using IRB-approved email scripts, information about the study and the contact information of a research team member. Participants were also asked to share or forward the email to other colleagues at their or other HIV/AIDS service organizations.

Based on previous research, we estimated reaching saturation after 12–15 interviews [34], thus our target enrollment was 15 participants, aiming for at least one interview per each of the 12 major HIV/AIDS service organizations in Indiana. In total, 15 semi-structured interviews were conducted with individuals from 10 of the 12 different HIV/AIDS service organizations across Indiana that focus on providing direct services to PLWH in Indiana. These organizations have multiple satellite locations and are well-dispersed geographically around the state and offer a diverse array of services including PrEP, non-medical case management, medical case management, HIV testing, STI testing, Hepatitis C testing, housing services, educational services, and mental health services – among others. The individuals interviewed held various roles within their respective organizations, including executive director, non-medical case manager, and testing coordinator.

Interviews of 30–60 minutes were performed virtually by a trained undergraduate male research assistant (JJM) with no current or previous associations with any of the interviewed individuals or organizations. The purpose of the study and the voluntary nature of their requested participation was explained to participants, they were given an opportunity to ask questions, and all participants provided verbal consent prior to commencing the interviews.

The interviews were conducted via video calling software (Zoom), recorded, and transcribed verbatim using Otter.ai. All interview transcripts were reviewed and edited for accuracy by two independent researchers. A codebook was developed based on the interview guide, initial reading of transcripts, existing literature, and guided by our theoretical framework (SEM). The transcripts were then thematically analyzed by two independent coders using a constant comparative method in NVivo, a qualitative coding software program. The coders first conducted open coding, where keywords and phrases that were included in the codebook were assigned to interview sections, followed by axial coding, where patterns between and within interviews were mapped to identify emerging themes [35]. Guided by the SEM, these themes were organized across individual-, interpersonal-, organizational-, community-, and public policy-levels. (Table 1). This study was exempt by Purdue University’s Institutional Review Board (protocol IRB-2020-685).

**Table 1:** Multilevel challenges and responses to supporting PLWH during the COVID-19 pandemic, based on the socio-ecological model framework

Individual	Interpersonal	Organizational	Community	Public Policy
• High COVID-19 risk perception • Pre-existing mental health conditions exacerbated • Decreased medication compliance and lack of recent lab results • Employment instability • Return to care with increased free-time due to unemployment	• Isolation from fellow PLWH • Resentment towards service providers due to in-person service restrictions	• Preparedness from experience with previous HIV outbreaks • New intakes and face-to-face interactions severely limited • Virtual intakes and follow-ups • Geographical and technological contact limitations with clients • Tablets for telehealth • No-contact home visits and at-will pickup for supplies and medications • Rental assistance • Initial decrease in HIV testing • Implemented self-swab testing, outdoor testing, and physical barriers	• Local private sector financial and material assistance • Collaboration with jails and treatment centers for HIV test kits and virtual instruction and education	• In-person requirement for quarterly review suspended • Laptop restrictions for work-related activity suspended • Sufficient CARES Act funding for clients • Lack of funding for operating expenses

## Results

### Individual-level challenges experienced by PLWH

According to organizational staff, PLWH were generally under the impression that they had an increased mortality risk upon contracting COVID-19, stating *“people living with HIV I think are especially aware of their compromised immune system. And so people are nervous to be out … I had one guy call and he said I’m scared to go out. I don’t have gloves. I don’t have a mask … I shouldn’t even go get no gas because of the pumps and stuff.”* This – at times – voluntary lack of facetime with close acquaintances or even strangers due to known health risks was coupled with an additional lack of *“intimacy and socialization”* available at service organizations because regular inter-client fraternization was prohibited due to public health restrictions: *“yes they got to see [service providers] but they never got to see their peers, like before when they were in the office they got to see each other, and that wasn’t happening when they were home.”* Even virtual contact with service providers was limited due to geographic and technological limitations, *“so I live in Brown County. We have great trees and lots of hills, also a good chunk of it is country. You just don’t have internet, like not because you can’t afford it, it’s just not available to you … So we did run into that with some clients and so there were some home visits in terms of like, I’m just gonna run out and check on someone.”*

Both knowledge of their HIV status and forced closures fostered feelings of isolation among PLWH. Moreover, pre-existing mental health issues were often aggravated by the additional isolation, with a counselor noting that *“they were just very anxious because a lot of them have anxiety already and they were just like what’s going on?”* Staff attributed to PLWH comments regarding a desire for in-person interaction, “*I wish I could hug you … and we have to say I’m sorry we can’t do that.”* At times, the inability to provide in-person services fostered resentment, *“so it was really hard for that change I think when we had to put a sign on our door that said no public allowed. And our clients couldn’t come in and we still have a client who like refuses to speak to us because he said that we’ve banned him from his home.”* In some instances, staff perceived that a lack of in-person contact could impact HIV medication adherence and thus impact viral suppression. For example, *“engagement and care really suffered … there are some of our clients that if they don’t have somebody like filling their pill boxes and monitoring it and checking on it, they’re just not gonna stay adherent to their medication.”*

Providers also suggested that employment cuts during COVID-19 also heavily affected their client population because *“most of our clients who are working work in the service industry … you know, there isn’t work. And so, certainly at any point in time, losing your housing is not going to be good for your overall health outcomes.”*

While some organizations noted a decrease in engagement from existing clients stemming from new onset or exacerbated mental health issues or job loss, others found that pandemic-induced unemployment allowed some individuals an opportunity to access services as some organizations observed new enrollees who had recently become unemployed: *“people were all off work. So they had time to take care of the medical stuff, you know. Sometimes when you’re working, because you’re going back and forth to work, you don’t take care of your own personal stuff. So we had three people who enrolled in our program, because they were off of work and they had the time to do it.”*

### Organizational-level challenges, and responses

#### Preparedness

A general theme among interviewed HIV service organizations about addressing issues raised by the COVID-19 pandemic was utilizing ingrained disaster and epidemic response techniques learned from dealing with the HIV crisis in the past because *“you think about back in the late 70s early 80s when AIDS hit, this country was woefully unprepared.”* Yet, as was the case across many different contexts, explicit preparation plans for an airborne, respiratory-based pathogen were nonexistent, *“it was pretty much doing everything on the fly. We have like a safety manual, but we didn’t have anything about a pandemic in there. And so definitely on the fly,”* so organizations had to and did act swiftly to properly address urgent, upcoming needs for both their clients and the PLWH population as a whole.

One organization reported preparing extensively for the pandemic while it was in its infancy, *“So we did an assessment. I want to say like the last week of February, first week of March, maybe, where our care coordinators contacted every client in every one of our programs to find out if they had what they needed to be able to shelter in place for 14 days. So that was a huge undertaking, and then we bought stuff.”* After the initial items and supplies had been purchased, they were compiled, organized, and distributed via no-contact *“home visits.”* Moreover, because of longitudinal relationships and familiarity with existing clients, staff members were able to identify clients that were particularly at risk for prescription non-compliance, *“we could identify like these are the people that I do a lot of medication management with. So we know if we’re not doing that we know they’re not taking them … whether we can document it or not, I know they’re not taking them.”* Consequently, staff ensured these individuals were able to receive and adhere to taking their medications by having their medications shipped to the office and communicating to clients *“alright your meds are here, I’m going to hand out the pillbox, they go back to their car, they fill the pillbox, they go in and hand it back, so you just, you know, you make it work.”* Multiple organizations also educated their clients on likely scenarios going forward, *“we had been able to let most of our clients know like, this is what’s happening with COVID and it is highly likely that Indiana will be on a stay at home order.”*

Additionally, because of the reciprocal nature of housing instability and infection, many organizations attempted to preemptively protect PLWH by assisting with their housing. For a period of time, preventing potential new instances of homelessness supplanted the shifting of existing clients around the housing case management network, with one organization stating *“we did very little of our normal like more comprehensive housing case management. It was very much crisis response for that entire time period like doing the application getting the documentation. Getting bills paid was pretty much what they were doing.”* Much of this effort was made in an attempt to mitigate the effects of lifting finite eviction moratoria, working with clients during the moratoria to avoid the need for a large amount of urgent aid, *“we went ahead and tried to help people along the way, because we knew the second that it was lifted, people were going to be facing eviction. So we tried to work with clients to prepare as if there wasn’t a hold on evictions.”*

Although long-term or permanent supportive housing programs may not have been the focus at the time, service providers did offer housing assistance and solutions to the newly homeless as well, *“we made sure to put them directly into some type of housing whether we put them in a hotel, or we found an apartment to put them in,”* although *“there [was] a lack of housing, rental housing. And if you [could] find rental housing, it [cost] was extremely high.”*

#### Outreach and testing

The COVID-19 pandemic severely affected the medium from which community-based organizations (CBO) interfaced with potential and existing clients. Many organizations stressed that they follow a harm reduction model and meet the client *“where they’re at, no judgement,”* indeed at times *“meet [ing] the people directly on the street.”* Organizations emphasized that they prefer face-to-face meetings, especially with initial intakes to pacify the nerves of new clients, *“I would like to think that this should be done in person at first, if at all possible … the person that’s coming in as a consumer is meeting you. They’re scared to death. They don’t know what to expect. They need to know who you are.”*

Prior to COVID-19, organizations made significant efforts building rapport with the communities with which they engage in an effort to better attract and retain clients: *“they trust us in a way that they won’t trust other places … rapport is an important part of retention and maintenance.”* In the past, having this level of rapport enabled CBOs to engage potentially at-risk clients, stating *“when we’re out on our mobile unit for our syringe exchange, we’ll go find somebody if we hadn’t seen him in a while just to check on and make sure they’re doing okay, why haven’t we seen you.”*

With the onset of COVID-19 restrictions, however, new intakes and face-to-face interactions were severely limited, with providers reporting *“we did not have new intakes for like a good month period … there was a solid several months there where people didn’t do health care in person at all”* and *“there were things that we did do in person, but I could probably count them on one hand.”*

Most organizations reported HIV testing significantly decreased during periods where in-person access to existing and potential clients was suspended or severely limited. Prior to COVID-19, HIV providers would typically test *“out in the community”* or *“every three months we were testing certain people especially at our drug treatment facilities … or the jail”* which comprised *“a significant portion of our HIV testing right there.”* Testing at these external sites, however, was no longer possible with public health restrictions in place. Moreover, the space limitations of testing facilities – *“physically our testing rooms were tiny”* – and the physical requirements of common testing modalities– *“we were doing fingerpick pricks at the time so you had to be pretty close to people”* – prevented adherence to safe public health practices.

Additionally, it was suggested that a decrease in HIV testing was also linked to COVID-induced changes in procedure. Once limited in-house testing resumed, one organization noted that their STD testing increased significantly from baseline while their HIV testing decreased significantly from baseline. It was hypothesized that these trends were observed because the STD testing underwent no location or procedural changes while location of HIV testing – previously out in the community – was forced to move in-house: *“our STD testing has always been done in house, so everybody knew that if you wanted STD testing you had to come to us we weren’t going to be in the community doing that … now for HIV they were not, they just knew that we were going to be in the community somewhere and if you found us you can get an HIV test. They were never trained to come to our office, or to sign up online like they were for STD.”*

To attempt to remedy the gap in testing due to social distancing, one service provider specified that they obtained more self-swab tests to limit client-provider contact, “*people could swab themselves. So you could sit farther or they could do it themselves and then you just look at it together in 20 minutes.”* Another provider further obtained a customized physical barrier between the tester and client: *“protection shields, so the client could stick because the test is doing a finger prick. So they could slide the hand through the shield. And so both people were protected.”* Other providers decided to eschew indoor testing altogether and maintained that outdoor testing would be most feasible and beneficial, *“so we do testing in the park, instead of testing an office or education at sites so people can just show up at a park and get tested for two hours at a time.”* To gain access to hard-to-reach populations in jails or vulnerable populations in treatment centers during in-person restrictions, one organization arranged special accommodations with such places, setting up zoom calls to undergo virtual testing and education: *“we’ll be zooming and then they’ll be referring people to us for testing, or we’ll be dropping off test kits at the facilities, and we’ll be zooming with them to do the testing after we do the education.”*

Although the pace of intakes initially slowed because of in-person outreach and testing restrictions, providers were eventually able to acquire the appropriate technology to perform virtual intakes and utilized basic phone calls and texting to obtain patient information: *“intakes [were] mostly done over the phone … we just asked people hey you know if you want we could get some basic information from you right now … and they can text us pictures of those things, they can email it, they can drop it off the office, we can go pick it up, we can do, like, what I call, like a door dash.”* Because of the newfound importance of connection via the telephone – for both interactions with the service organization and outside entities in a now predominantly virtual world – some organizations provided clients with funds to pay their telephone bill: “*that was their form of communication … So that was about 20 people we assisted who had lost their jobs to keep their cell phone on for a month, we thought it would just be a month. But it turned out, we had to do that a couple of times because people were not able to go back to work.”*

To assist both new and existing clients in accessing necessary case management services, many HIV service organizations provided select clients with tablets: *“for those who don’t have phones, or some people whose phones only work on Wi Fi, we’re able to distribute [tablets]. So people can do telehealth appointments, mental health appointments, anything with our programming, they’ll be able to do it from their tablet.”*

In regard to distributing goods to clients intermittently throughout the pandemic, organizations offered at-will pick up pursuant to social distancing guidelines, *“so right now, anybody who needs the supplies that we offer we have to just have them come and stand outside our door and give it to them or we can drop them off like there’s a table, and we just usually drop goods off there. And then, anybody can get it when they want.”* The distribution of supplies was not only as needed but proactive as well, placing provisions in highly trafficked areas, “w*e’ve also resorted to just setting up pockets of supplies inside gas stations, hotels, things where we know that people use intravenous drugs are. And our pamphlets we’ve put in local community centers so that they can just hand them out to patients that they know may need them.”*

### Public policy-level responses

Many organizations expressed the sentiment that, prior to the COVID-19 pandemic, their operations were forced to comply with unnecessary rules and restrictions that inhibited client interactions. Two particular policies were highlighted: the need to meet in person once every 90 days for a review, and the inability of organizations to use laptops for work-related purposes. Organizational staff found the quarterly in-person review to be a hindrance to both parties: *“So, what, what’s with all these different rules, you’d have to meet in person once every 90 days. What for? Whose needs does that serve? Not mine not theirs.”* Fortunately, this in-person requirement was suspended by the State of Indiana during the pandemic, and staff were able to sign on their client’s behalf. HIV service organizations appreciated this change and its added convenience *“if somebody doesn’t want to come in and meet with or doesn’t have the time but they can talk to us over the phone and give us all the information and then text me a picture of their ID … we can make things easier on people than we are.”* Moreover, proxy signings and virtual meetings not only increased convenience but also served to mitigate unnecessary disease propagation by resulting in *“a lot less actual contact with people.”*

In addition to the in-person review, the state-mandated restriction on laptop use hampered the ability of organizations to rapidly adjust to virtual work. Prior to the COVID-19 pandemic, Indiana was concerned about the security of laptops, *“the State Department of Health had never let us even own laptops before this, they considered it a security risk and information risk like even in our office they made us get desktop computers which are insanely more expensive than laptops … somebody could get [a laptop] stolen.”* Although organizations were not able to obtain laptops prior to COVID, many used cell phones to communicate with clients easily and remotely, *“we had work cell phones because we’ve gotten those a while back to be able to text with clients because we found a lot of clients preferred it. It’s just the way the world is now. But none of us have a laptop.”* Finally, the State eased restrictions and allowed HIV service organizations to use laptops for work-related affairs, *“and then all of a sudden everybody’s rules are just gone. Yes, it’s fine get laptops, work from home, set up remotely.”*

In terms of receiving financial support during the pandemic, organizations were able to receive a sufficient amount of funds able to be used for various client-facing initiatives: *“We have been fortunate to receive many COVID housing awards. So we have the funds to be able to put people in housing … we did get relief money from the CARES Act for both HOPWA and Ryan White Part B*^1^*, and we are putting that into our emergency financial assistance.”* Moreover, financial and material assistance did not just come from governmental aid but also the private sector as well, *“I think the Health Foundation of greater Indianapolis … I think they’re the ones who ended up paying for the laptops … The St. Joseph Community Health Foundation who funds I think general operating for us, they offered some up for like masks, sanitizers, cleaners, and things.”* Although the consensus seemed to be that an ample amount of funding was readily accessible for client-oriented programs, some organizations were concerned about the immediate and long-term availability of monies to sustain basic operating costs such as payroll and overhead. Operating funds were hypothesized to be reduced for two main reasons: a COVID-induced lack of in-person fundraising events and restrictions on the use-cases of existing funds: *“I think the place we’re gonna end up struggling is just general operating funds, because both of our big fundraisers for the year are having to be done virtually, so they’re not canceled, but they’re not going to be as productive as they typically are and that’s the thing nobody ever wants to fund is operating you know they want to give you money for housing and you know bus passes and things but they don’t want to pay for the person to hand them out to write the checks to process the paperwork.”* Going forward, organizations are appreciative of the immediate public and private sector responses but are concerned about their financial situation moving forward: *“So, yeah, the floodgates at the beginning were nice but I worry about the long haul.”*

## Discussion

COVID-19 has deepened inequalities and amplified social and structural determinants of HIV transmission. Global studies have highlighted various ways in which the COVID pandemic has impacted HIV testing and access to services for key populations. Data from the HIV modelling consortium suggests there may be a substantial rise in HIV infections and mortality from 2020 to 2025 due to COVID-related care disruptions if no additional support is provided to health systems and health workers [38]. This is compounded by poorer outcomes for both COVID and HIV for vulnerable populations such as minority ethnic, homeless, and PWUD populations, who will bear the brunt of weaker HIV services due to health disparities rooted in social determinants [39].

Despite the profound disruptions experienced by HIV programs, community-based HIV/AIDS service organizations responded quickly to the challenges posed by the COVID-19 pandemic and have adapted their service delivery practices to ensure that gains made in HIV prevention and management are not severely eroded. Studies have shown how community-based HIV programs made innovative service delivery adaptations to manage consequences of restrictions brought about by lock-downs including telehealth counseling, multi-month dispensing of ART, PrEP, needles and syringes and take home doses of opioid substitution therapy [40–43]. COVID has also shifted provider and public perceptions on innovations like HIV self-testing and community-based care delivery that move HIV diagnosis and management away from overburdened healthcare and laboratory facilities, which could have important implications for HIV care continuity and improve service delivery for marginalized populations long after the pandemic [44].

This study examined the impacts of the COVID-19 pandemic on PLWH in Indiana and the experiences, challenges, and responses of HIV/AIDS service organizations. The socio-ecological model guided the analysis of multilevel challenges and responses to maintaining HIV care during the pandemic, from the perspective of service providers. The identified individual-level challenges faced by PLWH support existing literature on the intersections between HIV and COVID-19 [8, 45, 46]. At the organizational level, Indiana HIV/AIDS service organizations reported numerous challenges and diverse responses to the COVID-19 crisis and impacts on their clients. While many HIV/AIDS service organizations had experience operating amid a viral epidemic, the transmissibility of COVID-19 limited any established disaster preparedness, presenting novel problems necessitating innovative solutions. As a result, HIV/AIDS service organizations utilized a motley of strategies to initiate and maintain care safely. HIV/AIDS service organizations elsewhere were also forced to suspend in-person HIV testing and various community outreach events [40, 41, 47]. Reponses elsewhere mirror those found in this study, including the pre-packing and delivery of various supplies as well as offering telephonic and virtual appointments, with the efficacy of the latter interventions varying by site [41, 43, 48]. Moreover, other organizations made additional attempts to mitigate the spread of COVID-19 at existing testing sites, such as pre-screening for signs of infection via the telephone or suspending HIV testing requirements to obtain PrEP services [47]. Overall, within Indiana and elsewhere, an initial suspension of in-person services was often supplemented with virtual equivalents, and – where appropriate – some in-person services such as testing were gradually reinstated pursuant to public health best practices at the time.

This study reveals findings that can be helpful to social service, public health, and medical practitioners providing HIV services to PLWH within their communities. The unique circumstances revolving around the COVID-19 pandemic, and the environment created, has led to a greater need for virtual healthcare services and advancements in telemedicine services provided to PLWH, specifically those residing in rural areas. Emerging research has demonstrated rural at-risk populations are amenable to the use of telemedicine for the provision of sexual health care and HIV service delivery [49]. Leveraging telemedicine beyond the COVID-19 pandemic period provides significant opportunities to enhance the HIV care continuum, specifically related to the prevention and treatment of HIV. The use of telemedicine, among PLWH, is an acceptable alternative to clinic visits and provides the ability to better fit appointments into a client’s schedule and decreased travel time to HIV clinics and service organizations.

Similarly, the implementation of at-home HIV screening during COVID-19 addressed access barriers in a time when clinics were closed. However, numerous barriers (e.g., stigma, privacy and confidentiality concerns, patient-provider relationships) will continue to prevent at-risk populations from accessing HIV and STI testing. Providing regular access to at-home tests, via mail order or pick up, is an opportunity to circumvent known barriers to uptake HIV/STI screening [50]. Piloting of at-home testing programs during COVID-19 has demonstrated increased demand from at-risk populations [51, 52]. The lack of in-person counseling in tandem with at-home HIV/STI screening is of potential concern. Future research is warranted on the delivery of sexual health interventions, such as the use of mobile health (mHealth), coupled with at-home testing.

This study and the conclusions drawn from it are not without limitations. As the interview participants were recruited only from Indiana, we cannot assume that our results are representative of the larger population of PWLH and HIV/AIDS service organizations in other states. Similarly, we relied on self-reported perceptions and experiences provided by participants in response to questions raised during the interview process. Self-report can reflect potential biases inherent in the use of interviews for data collection. These limitations could be overcome in future work by corroborating participant self-reports with deidentified client data on client healthcare utilization, HIV testing, and treatment compliance. Despite these potential limitations, our data provides much needed formative information on the impact of COVID-19 on HIV service delivery.

## Conclusions

The lessons provided by HIV/AIDS service organizations in Indiana are invaluable to informing HIV prevention and care continuity as well as future pandemic response for PLWH and at-risk populations. Furthermore, many ways in which they adapted their practices could improve service delivery for vulnerable, at-risk, and PLWH populations. Removing barriers, notably access and privacy concerns, to screening and treatment is one potential method to enhance the HIV care continuum beyond the COVID-19 pandemic.

## Supplementary Information

**Additional file 1.**

## Data Availability

The datasets used and analyzed during the current study are available from the corresponding author on reasonable request.

## Abbreviations

AIDS: Acquired immunodeficiency syndrome
COVID-19: Coronavirus Disease 2019
HIV: Human immunodeficiency virus
PLWH: People living with HIV
PrEP: Pre-exposure prophylaxis
PWUD: People who use drugs
SEM: Socioecological model

## Footnotes

1 The Housing Opportunities for Persons With AIDS (HOPWA) Program is a federally funded program that provides housing and other assistance for individuals diagnosed with HIV/AIDS that meet specific income requirements [36]. The Ryan White HIV/AIDS Program is a program that provides grants various entities including local service organizations to fund HIV healthcare and various support services [37].
